# Is It Important to Consider Sex and Gender in Neurocognitive Studies?

**DOI:** 10.3389/fpsyt.2015.00083

**Published:** 2015-06-02

**Authors:** Adrianna Mendrek

**Affiliations:** ^1^Department of Psychology, Bishop’s University, Sherbrooke, QC, Canada

**Keywords:** sex differences, gender, cognition and emotion, schizophrenia, drug addiction

Is it important to consider sex and gender in neurocognitive, neurobiological, and clinical studies? I refer here to “sex” as a biological variable related mainly to sex chromosomes and sex steroid hormones, while “gender” stands for a psychosociocultural construct related to gender role socialization, levels of masculinity and femininity, and stress related to adhesion to gender stereotypes. It is a rhetorical question and I hope that an overwhelming majority of readers would answer: “yes, of course!” However, despite the fact that more attention has been devoted over the past decade to delineating differences between men and women in their neuroanatomy, neurophysiology, and cognitive and emotional processing (1, 2), a substantial number of researchers (basic and clinical) continue ignoring “the second sex” and study exclusively males. The reasons are multiple and range from an unawareness, through a lack of sufficient funding to examine both sexes, to the argument that the studies, to date, have not found any significant sex differences in a given paradigm or disorder and therefore there is no need to include both males and females in a study. Finally, there is a group of theorists who sees this line of inquiry (i.e., investigating sex differences in the brain and cognition) as inherently biased, thus contributing to some harmful stereotypes that may lead to increasing gender inequalities [e.g., Ref. (3, 4)].

It is hard to argue with the lack of funds and indeed there are cognitive and clinical domains where we have not seen any indication of potential sex or gender differences. Nevertheless, our technology has improved and we possess more sensitive instrumentation, which may detect subtle differences that were not previously apparent. Moreover, we must remember that men and women of today are different from men and women of two or three decades ago. We are not only socialized differently, due to changed family values and education, more competitive job market and prominence of social media, but also exposed to more environmental toxins, including endocrine disruptors, which may affect our physical and mental health (5, 6). As to the harmful effects of some studies of the neurobiological and cognitive sex differences, I agree that data are sometimes interpreted in a biased manner and may contribute to propagation of damaging gender stereotypes. However, I also believe that excluding women is much more dangerous. We have seen the harm done with several drugs released for treatment of various disorders without proper testing or consideration of women’s physiology (e.g., Posicor, approved for the treatment of hypertension and angina, slowed or stopped the heart rate especially in elderly women; antihistamines such as Seldane and Hismanal induced cardiac arrhythmias disproportionally more frequently in women), but thankfully the situation is changing (7, 8).

While it is true that men and women are much more alike than different and it actually makes more sense to talk about sex and gender similarities (be it in brain structure or cognitive function), there are those subtle differences that may provide clues to disentangling etiology of some neurological and neuropsychiatric disorders, such as multiple sclerosis, autism, mood, and anxiety disorders, or shed a new light on treatment of these conditions. In short, “vive la similarité, but let’s explore differences!” I would like to use two clinical examples that I am most familiar with, to illustrate my point – schizophrenia and drug addiction.

## Schizophrenia

The existence of sex differences in the prevalence, development, and progression of schizophrenia was noted already by Kraepelin and Bleuler who conceptualized and meticulously described the disorder at the beginning of the twentieth century (9, 10). Possibly because the prevalence of schizophrenia is greater in men than in women during the first half of life (until about 40 years of age), most studies forgot about women altogether – this despite the fact that there is a second peak of new cases in women around the age of menopause when they catch up with men in the prevalence (11, 12). I was unaware of this bias when I started investigating neural correlates of cognitive function in schizophrenia during my graduate school years, but very soon afterwards, as an independent researcher, it became clear that existing theories and treatment of schizophrenia were based almost entirely on data from male subjects (human and animal). This realization hit me when I came across two intriguing research studies, which found a neuroanatomical reversal of normal sexual dimorphism in the anterior cingulate (13), amygdala, and orbitofrontal cortex (OFC) (14). Due to my research familiarity with functional magnetic resonance imaging (fMRI), I began to search for any fMRI studies of emotion processing in schizophrenia since the anterior cingulate, amygdala, and OFC are all part of the corticolimbic system heavily implicated in the experience and expression of affect. I found numerous studies that met my search criteria, but none of them examined sex differences as they included either exclusively men, or the number of women was too small to allow for any comparisons. This was almost a decade ago and it has motivated our group to make an extra effort to recruit women diagnosed with schizophrenia to our studies. We have conducted several fMRI studies; some revealed reversal of normal sexual dimorphism [e.g., during mental rotation task; (15)], others did not find any sex or sex-specific differences [e.g., during passive viewing of emotional stimuli; (16)], while a few found significant relationship between brain activations and sex steroid hormones [e.g., Ref. (17, 18)]. This line of research suggested to us that there might be a subtype of schizophrenia patients where symptoms and related brain dysfunction is partly fostered by hormonal imbalance during organizational and/or activational stage of neurodevelopment.

Thankfully, over the past decade the situation in the field has changed; sex differences in schizophrenia are more widely acknowledged and females (both animal and human) are more frequently studied or at least included in the protocols. It is also recognized that women might require lower doses of antipsychotic medications during their reproductive years (due to interactions between antipsychotic medications and estradiol) and that they may benefit from low doses of estradiol or selective estrogen receptor modulators (SERMs) (19). It is possible that factors contributing to the development of schizophrenia and related psychosis are slightly different in men and women or that these factors interact differently with the sexes. For example, perturbation in the organizational effects of testosterone *in utero* could affect male and female fetuses differently, and exposure to environmental toxins could affect endocrine systems of males and females differently.

## Drug Addiction

Drug addiction is another condition characterized by important differences between men and women, and demonstrates how research has changed over the past few decades. Somewhat similarly to schizophrenia, traditionally drug abuse and dependence have been considered a “male problem.” However, while the prevalence of alcohol and cannabis dependence is still greater among men, gender differences in the abuse of stimulants and prescription drugs seem to have disappeared in the Western world (20). In addition, women appear to be more prone to develop drug dependence, suffer more severe physical and psychological consequences of drug abuse, and have a more difficult time “kicking the habit” (21). The reasons for this gender gap include a mixture of biological and psychosocial factors. For example, while a larger proportion of men initiate drug use to induce feelings of elation, energy or focus, women frequently start taking drugs to alleviate pre-existing mental health problems, including depression and anxiety (22). This maladaptive self-medication strategy often results in a faster transition to a habitual drug use and eventually a more severe dependence (23, 24). In addition, the socio-cultural norms, particularly in the Western society, have changed dramatically over the past few decades. Thus, while there is still a more severe stigma and prejudice against women who use drugs, especially if they are pregnant or have children, overall there is greater acceptance of women’s drug use than it was several decades ago (25). Moreover, women have much greater access to various drugs of abuse than they used to have. Finally, over the past couple of decades, new research has suggested some neurobiological factors that could also contribute to sex differences in drug addiction. For example, there is evidence that the dopamine system, which for decades has been strongly implicated in drug reinforcement, is sexually dimorphic. The number of dopaminergic neurons, the density of the dopaminergic terminals, as well as responsiveness of the dopaminergic system to drugs of abuse, have been shown to differ between males and females and they have been shown to be modulated by sex steroid hormones, especially estrogen (22, 26, 27). All these psychological, socio-cultural, and biological factors that contribute to sex differences in drug use and drug dependence should be considered while evaluating and treating individuals with drug addiction problems.

Our research has focused specifically on addiction to nicotine partly because it is a significant problem in schizophrenia. In the general population, still more men than women smoke cigarettes, but this gap is decreasing steadily. Moreover, studies show that women become dependent faster and have more difficulties quitting the habit than men (28). The difficulty quitting and the higher relapse rates have been linked to greater levels of drug craving, although evidence is still equivocal. For example, we examined sex differences in cue-induced craving for cigarettes in non-deprived smokers and did not find any differences between men and women (29). There were, however, fluctuations in the craving-related fMRI activations across the menstrual cycle in women. I should highlight that in our study we tested only non-deprived smokers, while studies that have reported sex differences, typically assessed craving following a period of abstinence [e.g., Ref. (30, 31)]. Indeed, some studies suggest that men are more sensitive to cue-induced craving, while women react stronger to stress-induced craving [e.g., Ref. (32)]. These and similar studies may be helpful in developing gender-sensitive treatment programs.

I presented just two examples of neuropsychiatric problems where unraveling sex differences may benefit women and deepen our understanding of the disorders. For instance, several promising clinical trials have already been performed with low doses of estradiol to treat women and men with schizophrenia (19, 33). In terms of drug addiction, most rehabilitation programs are still based on a male model, but it is recognized that women may require additional support (e.g., family planning, childcare services) and approaches that emphasize stress reduction (34).

Before closing I would like to mention gender, femininity/masculinity and related variables, which have been almost entirely absent from the neuroscience research, with a few exceptions. For example, in one early study, Cahill et al. (35) demonstrated that although no differences were detected between sexes in emotional memory, when gender was taken into consideration, individuals with more masculine traits showed superior recall of central emotional information, whereas individuals with more feminine traits exhibited better recall of peripheral details. More recent neuroanatomical studies reveal comparable results. Thus, a study in healthy adults showed that identification with more feminine traits correlated with greater straight gyrus volume (part of the ventral frontal cortex) and with better performance on a social cognition task [interpersonal perception task; (36)]. A different study, in children, reported that higher masculinity predicted greater volumes of white matter in the frontal lobe, while higher femininity predicted greater volumes of gray matter in the temporal lobe (37). These studies point to the possibility that even though sex and gender are closely related, in some situations, gender differences may be more important than sex differences and thus both should be studied in human participants. However, we need better gender measures, as many tests and questionnaires, such as the popular Bem Sex Role Inventory (38) were developed in 1970s.

To conclude, neurocognitive, neurobiological, and clinical studies that do not include female participants (animal or human) present only half of the story, the male part. In some cases, this may not be a problem, but in many it can deter us from scientific progress in understanding etiology, and in developing successful treatments for neurological and neuropsychiatric disorders in men and women. Studies that do not include females are only half-truth, and as Cahill (39) so eloquently stated using a Yiddish proverb – “A half-truth is a whole lie.”

## Conflict of Interest Statement

The author declares that the research was conducted in the absence of any commercial or financial relationships that could be construed as a potential conflict of interest.
